# Safety Assessment of Microcatheter-Protected Rotational Atherectomy with the Double Guiding Catheter Technique for Severely Calcified Left Main Bifurcation

**DOI:** 10.1155/2022/1399510

**Published:** 2022-08-09

**Authors:** Shijun Yang, Silai Dong, Yanzhao Zhou, Yumiao Wei, Ning Zhao, Chunhua Sun, Xiang Cheng

**Affiliations:** Department of Cardiology, Union Hospital, Tongji Medical College, Huazhong University of Science and Technology, Wuhan 430022, China

## Abstract

**Background:**

Rotational atherectomy (RA) is a tool for calcium modification, but there is a risk of losing the side branch in left main coronary artery (LM) bifurcation lesions, resulting in disastrous consequences. Microcatheter-protected RA with the double guiding catheter (GC) technique for severely calcified LM bifurcations has been described previously, but its safety warrants further investigation.

**Methods:**

Various sizes of coronary calcification vascular simulators were utilized to model calcified LM bifurcation lesions for RA in in vitro. The damage to the side branch protective microcatheters and guidewires was accessed after microcatheter-protected RA with the double GC technique. In clinical practice, microcatheter-protected RA with the double GC technique was carried out in two patients.

**Results:**

In vitro, none of the protective microcatheters or guidewires were completely fractured, although the majority of them were damaged to varying degrees. In clinical practice, we successfully carried out two cases of percutaneous coronary intervention for severely calcified LM bifurcation with microcatheter-protected RA using the double GC technique.

**Conclusion:**

RA of severely calcified LM bifurcation lesions may be successfully performed using microcatheter-protected RA with the double GC technique, potentially reducing the risk of side branch occlusion. Since majority of protective microcatheters or guidewires were damaged, there was still some risk, and it is recommended to use this technique only in highly selected patient population of severely calcified true (Medina 1, 1, 1) LM bifurcations.

## 1. Introduction

Percutaneous coronary intervention (PCI) of lesions at left main (LM) bifurcations is consistently deemed a technical challenge because of the high incidence of complications and inferior clinical outcomes compared to those of nonbifurcation lesions. Bifurcation coronary artery intervention may increase the risk of occlusion of a side branch due to the carinal shift while handling (via a balloon, atherectomy, or stent) the main branch [1]. Fujino et al. found that coronary bifurcation lesions with calcification predicted a higher risk of side branch occlusion [2]. Plaque modification with rotational atherectomy (RA) may reduce this risk, but the associated side branch compromise still needs to be considered [3]. As an alternative solution, microcatheter-protected RA with the double guiding catheter (GC) technique, which has been reported in the past [4], raises concerns about safety (especially the possibility of complete fracture of the protective microcatheter and guidewires). Thus, we applied this technique in vitro to confirm its safety and successfully performed the technique on two patients with severely calcified LM bifurcations.

## 2. Methods

### 2.1. Materials Preparation

Microcatheters (130 cm, Finecross, Terumo, Japan), 7-F ABU 3.5GC (APT Medical, China), coronary guidewire (Asahi Sion, ASAHI, Japan), 7-F EBU 3.5GC (Medtronic, USA), the extracorporeal coronary vascular simulator (Boston Scientific Corporation, USA), and the 330 cm long RotaWire (Boston Scientific Corporation, USA) were prepared.

### 2.2. In Vitro Experiment

**Figure 1:**
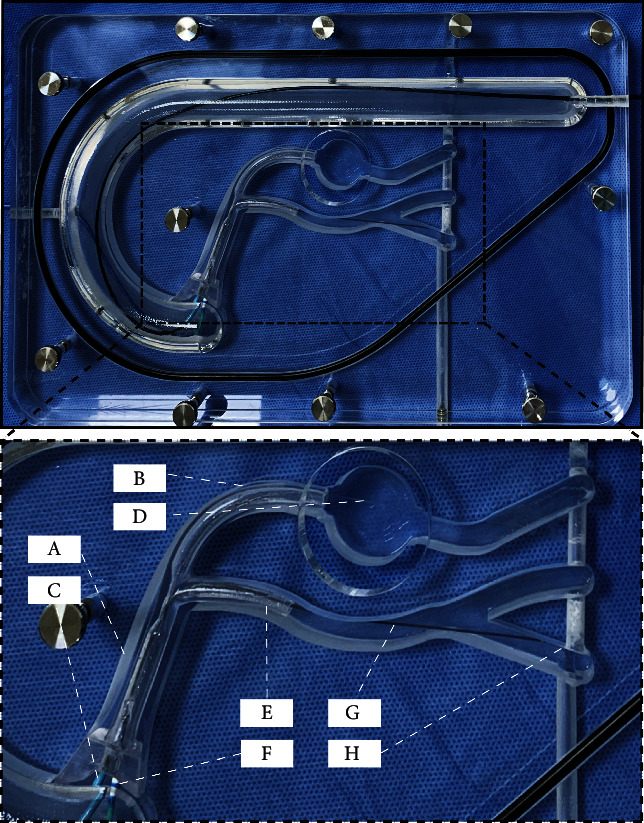
Extracorporeal coronary vascular simulator. (a) LM of the coronary vascular simulator. (b) LAD of the coronary vascular simulator. (c) 7-F ABU 3.5 GC (APT Medical, China). (d) 330 cm long RotaWire (Boston Scientific Corporation, Massachusetts). (e) LCX of the coronary vascular simulator. (f) 7-F EBU 3.5 GC (Medtronic, USA). (g) Coronary microcatheter (Finecross, Terumo, Japan). (h) 180 cm long coronary guidewire (Asahi Sion, ASAHI, Japan). LM, left main coronary artery; LAD, left anterior descending coronary artery; GC, guiding catheter; LCX, left circumflex coronary artery.

**Figure 2:**
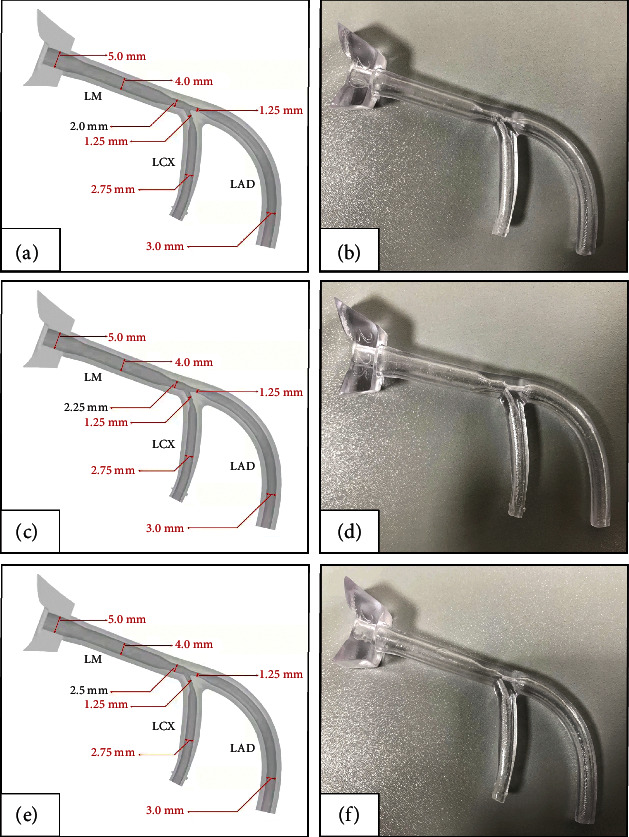
Structure diagram and real object of the coronary calcification vascular simulator with different diameters of distal LM. (a)-(b) The diameters of distal LM are 2.0 mm. (c)-(d) The diameters of distal LM are 2.25 mm. (e)-(f) The diameters of distal LM are 2.5 mm. LM, left main coronary artery; LAD, left anterior descending coronary artery; LCX, left circumflex coronary artery.

An extracorporeal coronary vascular simulator (Boston Scientific Corporation, USA, Figure 1) was used to simulate calcified lesions. Three sizes of coronary calcification vascular simulators (Figure 2) were used for microcatheter-protected RA with the double GC technique. The steps are as follows: A 7-F ABU 3.5 GC (APT Medical, China) was used to engage the LM. A 180 cm long coronary guidewire (Asahi Sion, ASAHI, Japan) was inserted into the left anterior descending artery (LAD) and was then replaced by a 330 cm long RotaWire through a coronary microcatheter in the anticipation of RA. Next, a 7-F EBU 3.5GC (Medtronic, USA) was used to engage the LM. A 180 cm long coronary guidewire with a 130 cm long microcatheter (protective microcatheter) was placed into the left circumflex artery (LCX) through the second GC. The RA of the LM-LAD axis was performed with a 1.25 mm burr while protecting the LCX with the microcatheter (Figure 1, Supplementary Video 1). When RA of the LM-LCX axis was performed with a 1.25 mm burr, the microcatheter was placed in the LAD to protect the LAD in the same manner (Supplementary Video 2). The damage to the side branch protective microcatheter and guidewire was evaluated after RA from the differently sized calcified lesion simulators. Observation and research indicators were as follows: number of damaged microcatheters, number of damaged guidewires, and number of completely fractured microcatheters or guidewires.

### 2.3. Clinical Practice

#### 2.3.1. Patient 1

**Figure 3:**
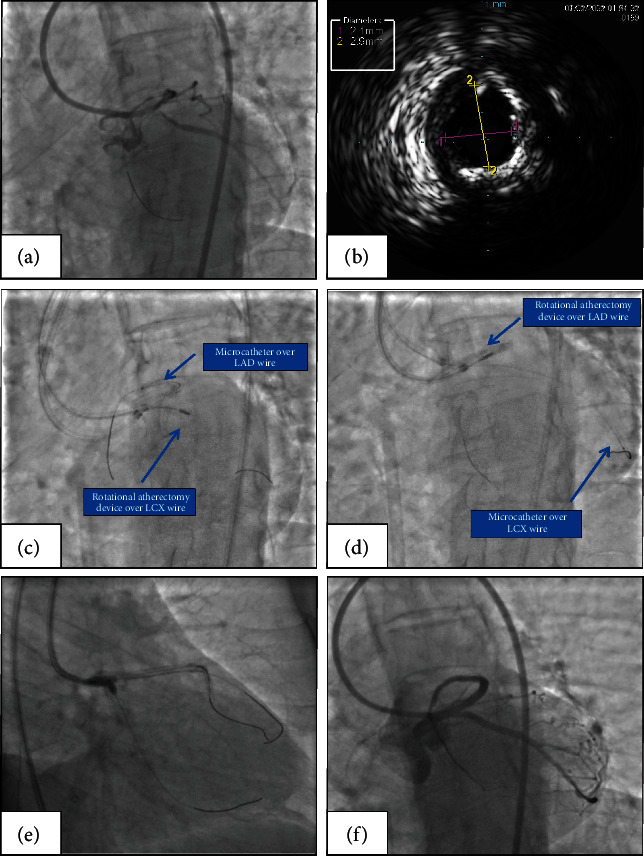
Microcatheter-protected RA with the double GC in patient 1 (a) Left coronary angiography revealing severe calcified LM bifurcation stenosis in LAO-caudal view. (b) IVUS showing severe calcification in distal LM. (c) RA performed on the LM-LCX axis via the first GC (7-F ABU 3.5 GC) with a 1.25 mm burr and the LAD protected by the microcatheter and guidewire through the second GC (7-F ABU 3.0 GC). (d) RA performed on the LM-LAD axis via the first GC (7-F ABU 3.5 GC) with a 1.25 mm burr and the LCX protected by the microcatheter and guidewire through the second GC (7-F ABU 3.0 GC). (e) Final kissing balloon with noncompliant balloons to optimize the LM reconstruction. (f) Final postintervention coronary angiography in LAO-caudal view. RA, rotational atherectomy; GC, guiding catheter; LAO, left anterior oblique; IVUS, intravascular ultrasound; LM, left main coronary artery; LCX, left circumflex coronary artery; LAD, left anterior descending coronary artery.

A 75-year-old female with diabetes mellitus and hypertension presented with a 2-week history of chest tightness on exertion. Coronary angiography revealed severely calcified bifurcation stenosis of the LM, LAD, and LCX coronary arteries (Figure 3(a), Supplementary Video 3). The patient refused to undergo cardiac surgery, and PCI was performed as an alternative treatment.

A 7-F ABU 3.5GC (APT Medical, China) was used to engage the LM. Two 180 cm long coronary guidewires were inserted into the LAD and LCX. Intravascular ultrasound (IVUS) was used to detect the LAD, LCX, and LM. IVUS showed calcification throughout the whole course of the LM (Figure 3(b)). Using a 130 cm long coronary microcatheter (Finecross, Terumo, Japan), the coronary guidewire in the LCX was replaced with a 330 cm long RotaWire (Boston Scientific Corporation, Massachusetts) in anticipation of RA. Then, a 7-F ABU 3.0 GC (APT Medical, China) was used to engage the LM. The protective microcatheter and guidewire were placed in the dominant LAD through the second GC. The RA of the LM-LCX axis was performed with a 1.25 mm burr while protecting the LAD with the microcatheter (Figure 3(c), Supplementary Video 4). Then, the microcatheter was removed from the LAD and advanced over the LCX guidewire into the LCX. The RA of the LM-LAD axis was performed with a 1.25 mm burr (Supplementary Video 5) and a 1.75 mm burr in the same manner (Figure 3(d)). After changing the guidewire, bifurcation intervention was performed using a double kissing crush (DK crush) technique to treat the LM, LAD, and LCX (Figures 3(e) and 3(f)).

#### 2.3.2. Patient 2

**Figure 4:**
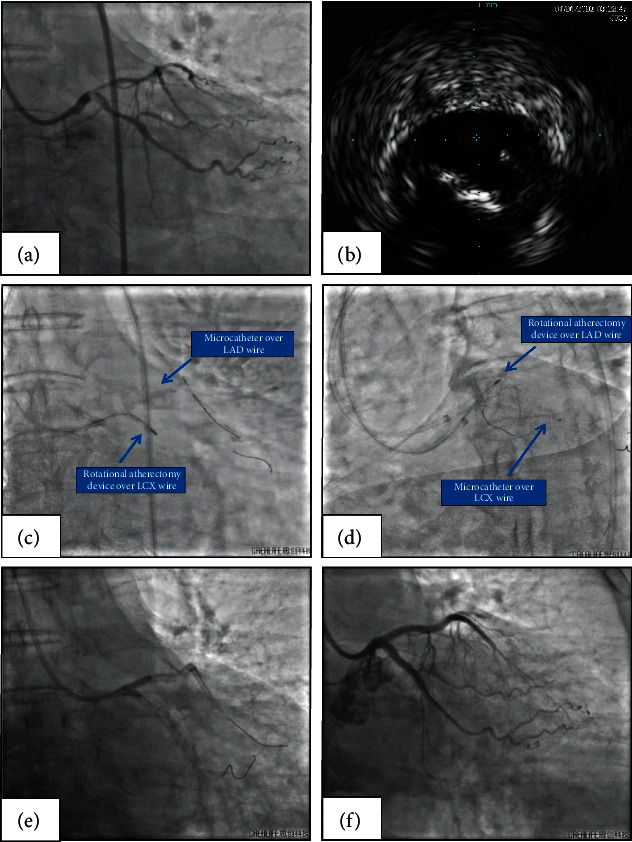
Microcatheter-protected RA with the double GC in patient 2 (a) Left coronary angiography revealing severe calcified LM bifurcation stenosis in caudal view. (b) IVUS showing severe calcified nodule at the distal LM. (c) RA performed on the LM-LCX axis via the first GC (7-F ABU 3.75 GC) with a 1.25 mm burr and the LAD protected by the microcatheter and guidewire through the second GC (7-F ABU 3.5 GC). (d) RA performed on the LM-LAD axis via the first GC (7-F ABU 3.75 GC) with a 1.25 mm burr and the LCX protected by the microcatheter and guidewire through the second GC (7-F ABU 3.5 GC). (e) Final kissing balloon with noncompliant balloons to optimize the LM reconstruction. (f) Final postintervention coronary angiography in caudal view. RA, rotational atherectomy; GC, guiding catheter; IVUS, intravascular ultrasound; LM, left main coronary artery; LCX, left circumflex coronary artery; LAD, left anterior descending coronary artery; RAO, right anterior oblique.

A 77-year-old male with diabetes mellitus and hypertension presented with angina on exertion, which he had experienced for 4 months. Coronary angiography revealed a severely calcified bifurcation lesion at the end of the LM, with involvement of the ostia of both the LAD and LCX (Figure 4(a), Supplementary Video 6). The patient strongly refused coronary artery bypass grafting and requested percutaneous coronary intervention.

A 7-F ABU 3.75GC (APT Medical, China) and a 7-F ABU 3.5GC (APT Medical, China) were used to engage the LM. A 180 cm long coronary guidewire was passed into the LAD and LCX through different GCs. IVUS was used to detect the LAD, LCX, and LM. The IVUS probe could not enter the LAD and LCX because of the severe calcified nodule at the distal LM (Figure 4(b)). Using a 130 cm long coronary microcatheter (Finecross, Terumo, Japan), the guidewire in the LCX was replaced with a 330 cm long RotaWire (Boston Scientific Corporation, Massachusetts) in anticipation of RA through ABU 3.75GC. Then, the protective microcatheter and guidewire were placed in the dominant LAD through the ABU 3.5 GC. RA of the LM-LCX axis was performed with a 1.25 mm burr while protecting the LAD with the microcatheter (Figure 4(c), Supplementary Video 7). The microcatheter was removed from the LAD and advanced over the LCX guidewire into the LCX. The RA of the LM-LAD axis was performed with a 1.25 mm burr (Supplementary Video 8) and a 2.0 mm burr in the same manner (Figure 4(d)). After changing the guidewire, bifurcation intervention was performed using a DK crush technique to treat the LM, LAD, and LCX (Figures 4(e) and 4(f)).

### 2.4. Ethical Approval

The study was approved by the Institutional Ethics Committees of Union Hospital, Tongji Medical College, Huazhong University of Science and Technology. The approval number was 20210731.

### 2.5. Statistical Analysis

SPSS version 15.0 (SPSS Inc., Chicago, IL, USA) for Windows was used for statistical analyses. Categorical variables are expressed as numbers and frequencies. Differences in categorical variables were measured by the chi-square test. *P* < 0.05 was accepted as a significant difference in the analyses.

## 3. Result

### 3.1. In Vitro Experiments

**Figure 5:**
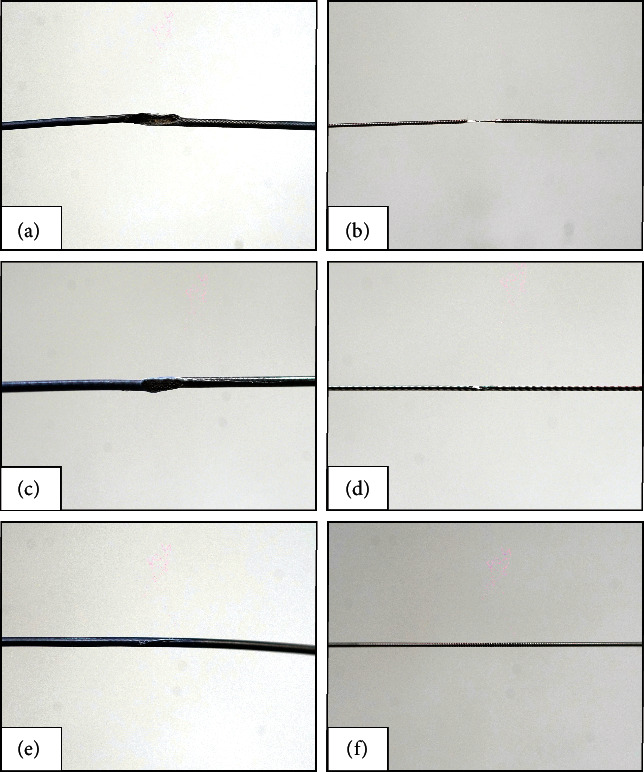
The representative damage of the microcatheters and guidewires after RA of vascular simulator with different diameters of distal LM. (a)-(b) The microcatheter and guidewire after RA of the 2.0LM vascular stimulator. (c)-(d) The microcatheter and guidewire after RA of the 2.25 LM vascular stimulator. (e, f) The microcatheter and guidewire after RA of the 2.5 LM vascular stimulator. RA, rotational atherectomy; LM, left main coronary artery; 2.0 LM/2.25LM/2.5LM, the diameter of the distal LM of vascular simulators was 2.0 mm/2.25 mm/2.5 mm.

All the RA burrs successfully passed through the simulated calcified vascular stenosis. Most of the microcatheters (88.9%, 16 of 18) and guidewires (77.8%, 14 of 18) in the side branch were damaged after RA (Table 1). There was no significant difference in the probability of damage to the microcatheter or guidewire used to protect the LCX and LAD. The damage rates of the protective microcatheters (66.7%, 4 of 6) or guidewires (33.3%, 2 of 6) in the 2.5 LM group were obviously lower than those in the <2.5 LM group (microcatheter: 100%, 12 of 12; guidewire: 100%, 12 of 12) (Table 2). This suggested that the large space of distal LM can avoid friction and damage to the protective microcatheter and guidewire when the RA burrs pass through. Although most microcatheters and guidewires were damaged to varying degrees, none of them was completely fractured (Figures 5, Tables 1 and 2).

### 3.2. Clinical Practice

**Figure 6:**
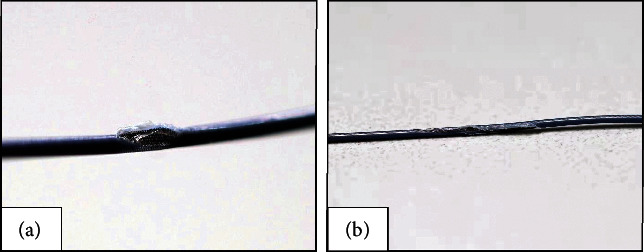
The damage condition of the microcatheters after RA in clinical practice. The microcatheter after RA of patient 1 (a) and patient 2 (b). RA, rotational atherectomy.

In clinical practice, we successfully carried out two cases of microcatheter-protected RA with the double GC technique for severely calcified LM bifurcation. The microcatheters of patient 1 and patient 2 were both damaged (Figure 6), but none of them were completely fractured.

## 4. Discussion

Acute occlusion of the LAD branch or LCX branch due to a carina or plaque shift will cause major adverse outcomes in PCI of true LM (Medina 1, 1, 1) bifurcation. It is known that the calcified coronary bifurcation lesions indicate a higher risk of side branch occlusion [2]. Therefore, side branch protection during PCI of LM calcified bifurcations has become mandatory. At present, there are several interventional treatments for coronary calcification, such as focused force dilatation balloons, scoring balloons, cutting balloons, rotational atherectomy, orbital atherectomy, and coronary intravascular lithotripsy [3]. Rotational atherectomy is a routine and efficient interventional treatment for coronary calcified lesions. It forms a new channel by plaque modification after polishing of the calcified plaque, which is convenient for the passage of subsequent instruments and conducive to the success of balloon expansion [5]. For bifurcation lesions, during the performance of RA, only the RotaWire should be placed in the target vessel to avoid cutting other guidewires and preventing guidewire entrapment with burr rotation [4, 6]. Unfortunately, some researchers have reported that the proportion of side branch occlusion caused by direct coronary RA is as high as 7.5% [7], which would be disastrous in the PCI of LM bifurcation. Protecting the side branch flow has become a difficult problem for cardiovascular interventional doctors.

Microcatheter-protected RA with the double GC technique is an effective method to protect the side branch during PCI of severely calcified LM bifurcations, which was first reported by Medda et al. in 2019. However, if the microcatheter or guidewire used to protect the side branch is completely fractured during RA, it might become an intravascular foreign body that can hardly be removed, with the occurrence of possible complications such as acute LM thrombosis. To avoid potential risk, we carried out an in vitro experiment to assess the safety of this method. The in vitro experimental results showed that although the side branch protective microcatheters and guidewires were damaged to varying degrees after RA, all the side branch protective microcatheters and guidewires were not completely fractured. On the other hand, the side branch protective microcatheters and guidewires in the 2.5 LM group had a smaller proportion of damage, suggesting that the large end space of the distal LM can reduce the friction and damage to the protective microcatheter and guidewire when the RA burr passes through.

The possibility that debris shed from the microcatheter and guidewire would increase the probability of no-reflow/slow reflow which cannot be totally ignored. Thus, considering the distinction between the real calcified human blood vessel and the vascular calcification simulator, measures should be prepared to handle situations in which the protective microcatheter or guidewire is completely fractured and becomes an intravascular foreign body in clinical practice.

## 5. Conclusion

The RA of severely calcified LM bifurcation lesions may be successfully performed using microcatheter-protected RA with the double GC technique, potentially reducing the risk of side branch occlusion. Since the majority of protective microcatheters or guidewires were damaged, there was still some risk, and it is recommended to use this technique only in highly selected patient population of severely calcified true (Medina 1, 1, 1) LM bifurcations.

## Figures and Tables

**Table 1:** In vitro rotational atherectomy outcome.

	RA of LM-LAD (*n* = 9)	RA of LM-LCX (*n* = 9)	*P* value
Microcatheter damage	8 (88.9%)	8 (88.9%)	1.000
Guidewire damage	8 (88.9%)	6 (66.7%)	0.257
Microcatheter completely fracture	0	0	
Guidewire completely fracture	0	0	

Data are presented as number (%); RA, rotational atherectomy; LM, left main coronary artery; LAD, left anterior descending coronary artery; LCX, left circumflex coronary artery.

**Table 2:** Outcome of in vitro rotational atherectomy with different LM sizes.

	RA of <2.5 LM (*n* = 12)	RA of 2.5 LM (*n* = 6)	*P* value
Microcatheter damage	12 (100%)	4 (66.7%)	0.034
Guidewire damage	12 (100%)	2 (33.3%)	0.001
Microcatheter completely fracture	0	0	
Guidewire completely fracture	0	0	

Data are presented as number (%). RA, rotational atherectomy; LM, left main coronary artery; <2.5 LM, the diameter of the distal LM of vascular simulators was less than 2.5 mm, which including 2.0 mm and 2.25 mm; 2.5 LM, the diameter of the distal LM of vascular simulators was 2.5 mm.

## Data Availability

The data used to support the findings of this study are included within the article and supplementary information file.

## Supplementary Materials

Supplementary Materials Video 1. RA of the LM-LAD axis was performed with a 1.25 mm burr in vitro. Video 2. RA of the LM-LCX axis was performed with a 1.25 mm burr in vitro. Video 3. Patient 1 coronary angiography in LAO-caudal view. Video 4. Patient 1 RA of the LM-LCX axis was performed with a 1.25 mm burr. Video 5. Patient 1 RA of the LM-LAD axis was performed with a 1.25 mm burr. Video 6. Patient 2 coronary angiography in caudal view. Video 7. Patient 2 RA of the LM-LCX axis was performed with a 1.25 mm burr. Video 8. Patient 2 RA of the LM-LAD axis was performed with a 1.25 mm burr. Supplementary videos can be viewed through the shared Google Drive link: https://drive.google.com/drive/folders/14ZTLcn_YtknxrzVwcRGzNnnHnpz8GNpj?usp=sharing.
