# Supplementary material for: Safety Assessment of Microcatheter-Protected Rotational Atherectomy with the Double Guiding Catheter Technique for Severely Calcified Left Main Bifurcation
Source: J Interv Cardiol. 2022 Aug 9;2022:1399510. doi: 10.1155/2022/1399510 (PMC9381278; doi:10.1155/2022/1399510)
Supplement: Supplementary Materials — Video 1. RA of the LM-LAD axis was performed with a 1.25 mm burr in vitro. Video 2. RA of the LM-LCX axis was performed with a 1.25 mm burr in vitro. Video 3. Patient 1 coronary angiography in LAO-caudal view. Video 4. Patient 1 RA of the LM-LCX axis was performed with a 1.25 mm burr. Video 5. Patient 1 RA of the LM-LAD axis was performed with a 1.25 mm burr. Video 6. Patient 2 coronary angiography in caudal view. Video 7. Patient 2 RA of the LM-LCX axis was performed with a 1.25 mm burr. Video 8. Patient 2 RA of the LM-LAD axis was performed with a 1.25 mm burr. Supplementary videos can be viewed through the shared Google Drive link: https://drive.google.com/drive/folders/14ZTLcn_YtknxrzVwcRGzNnnHnpz8GNpj?usp=sharing. [file 1399510.f1.docx]

Supplementary videos can be viewed through the shared Google Drive link：

https://drive.google.com/drive/folders/14ZTLcn_YtknxrzVwcRGzNnnHnpz8GNpj?usp=sharing
